# Link Scheduling Algorithm with Interference Prediction for Multiple Mobile WBANs

**DOI:** 10.3390/s17102231

**Published:** 2017-09-28

**Authors:** Thien T. T. Le, Sangman Moh

**Affiliations:** Department of Computer Engineering, Chosun University, 309 Pilmun-daero, Dong-gu, Gwangju 61452, Korea; thienle@chosun.kr

**Keywords:** wireless body area network, link scheduling, interference, interference prediction, mobility, medium access control

## Abstract

As wireless body area networks (WBANs) become a key element in electronic healthcare (e-healthcare) systems, the coexistence of multiple mobile WBANs is becoming an issue. The network performance is negatively affected by the unpredictable movement of the human body. In such an environment, inter-WBAN interference can be caused by the overlapping transmission range of nearby WBANs. We propose a link scheduling algorithm with interference prediction (LSIP) for multiple mobile WBANs, which allows multiple mobile WBANs to transmit at the same time without causing inter-WBAN interference. In the LSIP, a superframe includes the contention access phase using carrier sense multiple access with collision avoidance (CSMA/CA) and the scheduled phase using time division multiple access (TDMA) for non-interfering nodes and interfering nodes, respectively. For interference prediction, we define a parameter called interference duration as the duration during which disparate WBANs interfere with each other. The Bayesian model is used to estimate and classify the interference using a signal to interference plus noise ratio (SINR) and the number of neighboring WBANs. The simulation results show that the proposed LSIP algorithm improves the packet delivery ratio and throughput significantly with acceptable delay.

## 1. Introduction

In recent years, new technology allows wireless sensors to be placed in, on, or around the human body. A wireless body area network (WBAN) consists of a coordinator and several sensors that can be developed for medical and non-medical applications [1]. The coordinator of a WBAN is the controller that coordinates the communication between the coordinator and sensor nodes within the WBAN and also connects to the other networks. In most implementations, the coordinator of a WBAN is a personal digital assistant device or smartphone that collects vital information and then send to the health monitoring center [2]. The sensors can be placed on, around, or implanted in the human body to collect vital signals, and are capable of sending or receiving signals through wireless communication links [2,3]. With the development of wireless sensors for health monitoring, a person who wears the sensors can move freely inside a hospital or can even do daily activities at home. Therefore, health monitoring can become more practical and less costly for patients. Currently, the standardization of WBANs has been established by IEEE 802.15.4 [4] and IEEE 802.15.6 [5]. More specifically, the IEEE standard 802.15.6 includes recently developed physical and multiple access control (MAC) protocols for short-range wireless communication for sensors near, in, or on the human body. Furthermore, cognitive radio networks for the wireless body network have been investigated for electronic healthcare (e-healthcare) applications [6].

Unlike wireless sensor networks, WBANs are mobile due to the unpredictable movement of humans in public places such as hospitals, bus stations, or schools. Consequently, the performance of a WBAN can be degraded by the collision of concurrent transmissions of nearby WBANs. Therefore, while tackling the design issues of WBANs, it is necessary to consider the environment of coexisting WBANs. In the IEEE 802.15.6 standard, the coexistence of multiple WBANs includes static, dynamic, and semi-dynamic environments as shown in Table 1 [5]. In addition, mobility support in WBANs can be considered because the network topology changes frequently, according to the mobility of the human [7]. Investigations into the problems surrounding mobility in WBANs have considered the distance between WBANs, the topology change in multiple WBANs, and the received power. The Bayesian inference classifier is applied to predict the next coexistence state for multiple mobile WBANs by using signal to interference plus noise ratio (SINR) values and the value of the packet delivery ratio at the current state [8]. In addition, the interaction between WBANs has been investigated with regards to the probability of interference by taking into account the distance between coordinators in cyber-physical WBANs based on Bluetooth technology [9].

Because of the randomly distributed deployment and unpredictable movement, WBANs that share the same frequency channel can collide due to the concurrent transmission of two or more nearby WBANs [10,11]. As a result, inter-WBAN interference may occur with the significant loss of data or low quality transmission. For example, in the hospital scenario, if the coordinator cannot receive any emergency signals from on-body sensors, there is a loss in the feed of vital signals. Therefore, it is necessary to avoid interference to ensure the continuity and quality of the transmission. In addition, the new paradigm of body-to-body networks focuses on energy efficiency, quality of service (QoS), interference coexistence, and mobility prediction [12]. Furthermore, some interference mitigation techniques focus on controlling the transmission power, scheduling the transmission time in either time domain or frequency domain [13]. In addition, the coexistence of mmWave WBANs has been examined at 60 GHz using the game-theoretic approach of power control to maximize the signal-to-noise-plus-interference-ratio at each WBAN [14].

Many interference mitigation schemes and MAC protocols have been designed to mitigate node-level interference and WBAN-level interference [13,14,15]. Even though existing work has predicted the coexistence environment of WBANs and mitigated the inter-WBAN interference, designing the algorithm to predict interference in densely deployed WBANs is still a challenging issue. However, the movement of the human body is different from that in mobile ad hoc networks (MANETs) due to the group mobility of the coordinator and sensors within a WBAN. Therefore, it is necessary to consider the unexpected mobility in WBANs before developing an interference prediction algorithm for multiple mobile WBANs.

In this paper, a link scheduling algorithm with interference prediction (LSIP) for multiple mobile WBANs is proposed. First, the interference prediction mechanism based on the Bayesian inference classifier is developed using SINR values and the number of neighboring WBANs. Then, the link scheduling algorithm is proposed by exploiting superframe structure, common scheduling, and negotiation. The proposed LSIP allows multiple coexisting mobile WBANs to transmit simultaneously without causing inter-WBAN interference. With the LSIP, each superframe includes a contention access phase (CAP) and a scheduled phase (SP) for two different types of medium access mechanisms. That is, the CAP of a superframe is used for the transmission of signals for non-interfering sensors using carrier sense multiple access with collision avoidance (CSMA/CA), whereas the SP of a superframe is used for the transmission of signals of interfering sensors using time division multiple access (TDMA). According to our simulation results, the packet delivery ratio and network throughput of the proposed LSIP significantly improve with acceptable delay compared to that of the conventional scheme.

The remainder of this paper is organized as follows: In the following section, related works are reviewed focusing on interference mitigation and prediction for efficient link scheduling in coexisting WBANs. In Section 3, the interference prediction mechanism for multiple mobile WBANs is presented. In Section 4, the proposed LSIP algorithm including superframe structure, common scheduling, and negotiation is presented in detail. In Section 5, the performance of the proposed scheme is evaluated via a computer simulation and compared with the conventional scheme. Finally, the conclusions are given in Section 6.

## 2. Related Works

In this section, we review some relevant work which deals with efficient link scheduling for multiple mobile WBANs. There have been investigations into the effects of interference on network performance metrics such as packet delivery ratio, latency, and QoS. Interference between disparate WBANs as well as interference with other wireless systems should be reduced [15]. Recently, some interference mitigation techniques have been developed for multiple WBANs coexistence, resulting in increasing packet delivery ratio and decreasing latency [13]. In our previous work of interference mitigation, the scheduling algorithm improves the network performance regarding to the priority of the sensed data [16]. In [17], the algorithm exploits a hybrid MAC superframe that is comprised of CSMA/CA and TDMA to avoid concurrent transmission from nearby WBANs. Moreover, the coexistence of WBANs and wireless local area networks (WLANs) has been investigated in medical environments in terms of the packet error rate [18]. In [19], the set of the channel states of sensor nodes to the coordinator is defined as the collective channel state which is used as a factor in designing a scheduling algorithm. In addition, the scheduling algorithm also selects the collective buffer state, and the time index to determine the transmission of the next superframe by sensor nodes to ensure QoS in medical environments. Some other scheduling schemes focus on the transmission of multiple WBANs in healthcare sensor networks [20,21]. In [22], cooperative scheduling for WBANs applies horse racing scheduling for a single WBAN and considers multi-WBAN concurrent transmissions as a game. In addition, the authors in [23] created an optimization model for the coexistence of IEEE 802.11 and 802.15.4 standards. The power control game has been applied to WBANs to adjust the transmission power from sensors or coordinators in [24].

Because of mobility, it is necessary to predict the coexistence for WBANs and their associated interactions in the same vicinity. The authors in [8] developed an algorithm for coexistence prediction in WBANs, which considers the current status according to the defined coexistence environment in the IEEE 802.15.6 standard. The work in [9] detects the presence of nearby WBANs. Furthermore, there are mechanisms for mobility prediction in MANETs, which consider the duration that two nodes stay connected [25,26]. In contrast, the mobility of WBANs depends on the typically random group mobility of the sensors in the human body. Hence, the mobility prediction techniques of MANETs cannot be applied to predict the mobility of WBANs. However, the interference detection for mobile WBANs is considered in a QoS-based MAC protocol for WBANs before starting intra-WBAN transmission [27]. In [28], the adaptive CSMA/CA mechanism is applied at the coordinator to avoid collision, and it achieves good performance in terms of high throughput and low collision rates. Another MAC protocol also derives a TDMA schedule for multiple WBANs [29]. Thus, it is necessary to consider the group mobility of WBANs in the interference mitigation algorithm.

## 3. Interference Prediction for Mobile WBANS

### 3.1. Network Model

A multi-WBAN network consists of *n* mobile WBANs, denoted as *B_i_*, 1 < *i* < *n*, where each WBAN consists of one coordinator and *m* sensor nodes. There are two types of intra-WBAN communications: a sensor node sends data to the coordinator and vice versa. For simplicity, the transmission range of a WBAN is denoted as a circle with radius *R* in which intra-WBAN communication occurs. However, the inter-WBAN communication is defined as the transmission between multiple coordinators of WBANs in the same vicinity. In general, if one or more nodes are within the transmission range of a node, they are called neighbors of the node. In this paper, the term neighbor is used to describe the interfering source of a WBAN. In our study, the free space path loss model is assumed for intra-WBAN and inter-WBAN links. The channel gain of body-to-body links is modeled as gamma distribution as in [30].

At time *t*, γ*_i,s_*(*t*) is the SINR at the coordinator of *B_i_*, as the signal comes from sensor *s* in *B_i_* and it is defined as

(1) $$\gamma_{i,s}(t)=\frac{P_{i,s}(t)}{N_{0}+\sum_{j\neq i}P_{i,j}(t)},$$

where *P_i,s_*(*t*) and *P_i,j_*(*t*) are the received signal power at the coordinator of *B_i_* for the signals that originate at sensor *s* in *B_i_* and the received signal power from the coordinator of *B_j_*, respectively.

The free space path loss model uses the path loss exponent of 2 and is proportional to the distance between transmitter and receiver. The received power at the coordinator of *B_i_*, *P_rx_* is represented as

(2) $$P_{rx}(t)=P_{tx}\times Gain_{tx}\times Gain_{rx}\times {\left(\frac{\lambda }{4\pi d(t)}\right)}^{\eta },$$

where *P_tx_* is the transmit power, *η* is the path loss exponent, and *d*(*t*) is the distance from the source to the coordinator of *B_i_*; *d*(*t*) can be *d_i,s_*(*t*) or *d_i,j_*(*t*) for intra- or inter-WBAN transmission, respectively. 

Mobility is caused by human posture and movement which changes rapidly over time. The operating time can be divided into a set of *T* epochs. Therefore, at each time *t*, we consider multiple WBANs as a graph *G*(*t*) = (*V*(*t*), *E*(*t*)), in which *V*(*t*) is the set of WBANs at time *t* and *E*(*t*) is the set of interfering links between WBANs at time *t*. An interference link is defined if the transmission ranges of two nearby WBANs overlap. If the distance between two WBANs is less than the interference range, they interfere with one another. A WBAN *B_i_* is interfered with if any sensor interferes with other WBANs, or *γ_i_*(*t*) < *γ_th_* | *γ_i_*(*t*) = *min*{*γ_i,s_*(*t*), 1 ≤ *s* ≤ *m*}. The interference links at *B_i_* can be represented as *d_i,j_*(*t*) < *R* | *i,j* ∈ *V*(*t*)*.* The set of neighbors that interferes with *B_i_* is denoted as *NB_i_*(*t*) = {*NB_i_*(*t*) ∪ *B_j_*| *γ_i_*(*t*) < *γ_th_, d_i,j_*(*t*) < *R*}. The set of all interfering WBANs in the network is denoted as *S*(*t*) *=* {*NB_i_* (*t*) ∪ (*NB_j_* (*t*) | *I, j* ∈ *V*(*t*)}. The maximum degree of *S*(*t*) is denoted as Δ*_S_*_(*t*)_ = max{Δ(*B_i_*)| *i* ∈ *V*(*t*)} in which Δ(*B_i_*) is the degree of *B_i_*. Each *B_i_* creates interfered sensor groups and non-interfered sensor groups: *I_i_* = {*s* | *γ_s,i_* < *γ_th_,* 1 ≤ *s* ≤ *m*}, *NI_i_* = {*s* | *γ_s,i_* > *γ_th_*,1 ≤ *s* ≤ *m*}, respectively.

Because each WBAN is considered as a mobile network, the reference point group mobility model (RPGM) [26] can be used to represent WBAN movement behavior. For each WBAN, the coordinator is considered as the reference point in the group mobility model and moves with a constant speed *v_i_*(*t*) in each epoch, while all sensor nodes within the WBAN follow the coordinator’s motion behavior. The group motion vector of the coordinator will map the location of the coordinator whereas all sensor nodes add the node-dependent random motion vectors to the group motion vector. At time *t*, the location of the sensor node *Y_s_*(*t*) in a WBAN is given by the following: *Y_s_*(*t*) = *Y_c_*(*t*) + *RV_s,c_*(*t*), where *RV_s,c_*(*t*) is a node-dependent local displacement or random motion vector of the sensor nodes, and *Y_c_*(*t*) is the location of the coordinator at time *t*.

In a real-world scenario, people wearing a WBAN can move either close to or far away from another WBAN. We define interference duration at a WBAN as the duration that the WBAN is interfered with by the concurrent transmission of other WBANs. If the transmission ranges of two WBANs overlap for more than the threshold time, the interference duration is long; otherwise, the interference duration is short. In the case of short interference duration, one WBAN may move away from the overlapped transmission range.

**Figure 1:**
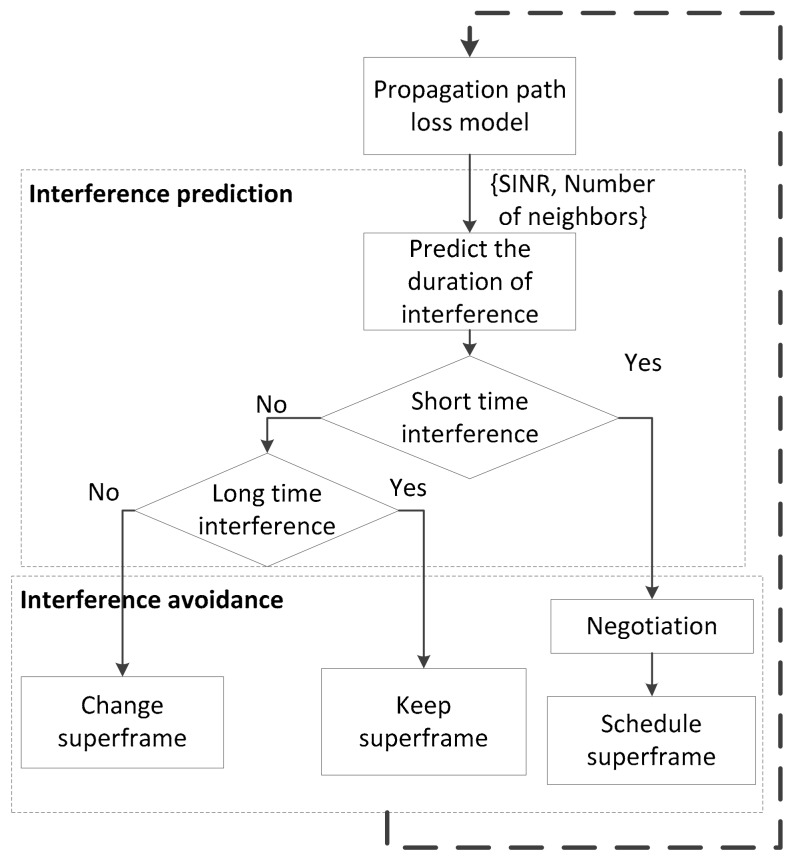
Interference prediction and avoidance at each WBAN coordinator.

For interference avoidance, we take into account the negotiation between the coordinators of interfered WBANs as follows: the coordinators will exchange the schedule during the negotiation time before starting a new superframe. We assume that all the coordinators are synchronized so that the superframe can start at the same time for all coordinators. Figure 1 shows the interference prediction and avoidance at each WBAN coordinator. In the LSIP, the interference avoidance step allows WBANs to use two different types of MAC superframes with regard to interference duration. In the case of long time interference, the number of neighbors may not change immediately. Hence, the schedule of multiple WBANs can be kept the same. However, if the number of neighbors is either increased or decreased, the schedule in the superframe may be changed. Due to the negotiation step and the scheduling algorithm, the continuity of signals from sensor nodes to the coordinator is ensured within the acceptable delay. However, the delay is constrained by the negotiation time between a WBAN and its neighbors, which must be below a given threshold. We propose a scheduling algorithm for multiple WBANs, which uses a hybrid MAC superframe structure based on the hybrid carrier multiple access of CSMA/CA and a scheduled part with TDMA. The coordinators will negotiate with the neighbor WBANs for the schedule of the nodes’ transmissions. By applying TDMA in the MAC superframe, the two-hop WBANs can reuse the time slot, which will increase the network throughput.

### 3.2. Bayesian Inference Classifier for Interference Prediction

The IEEE standard 802.15.6 has defined the coexistence environment of multiple WBANs in Table 1. The coexistence states are categorized by the duration of the coexistence condition based on the mobility level (i.e., slowly moving or fast moving) and the traffic data rate. However, the parameters for distinguishing between the states are not clearly defined in the standard.

In our algorithm, we consider the number of neighbors and the SINR value to obtain the duration of interference. The duration of interference is represented as *T_IF_*, which can be divided into two cases: short-time interference (*ShortIF*), if the interference duration is shorter than a given threshold value *T_thr_*, and long-time interference (*LongIF*), if the interfered duration is longer than *T_thr_*. Therefore, the state of the WBAN can be one of three cases: no interference (*None*), *ShortIF*, and *LongIF*. We apply the inference model of the Bayesian inference classifier in [31] to the interference prediction process to determine the next state of a WBAN. The Bayesian inference model is chosen because of low complexity so that it can be easily applied to WBANs with high accuracy.

The Bayesian inference classifier in [31] which operates as an inference system is used to predict the next state of a WBAN. The Bayesian inference classifier has two input values: the number of interfering neighbors and the SINR value. These are represented as *x_NI_* and *x_SINR_*, respectively. The possible state is defined as *State* = {*None*, *ShortIF*, *LongIF*}. The classifier will choose the most probable value among the possible values according to the input training values by using the maximum a posteriori (MAP) method. The probability of predicting the next state uses naïve Bayesian prediction. Hence, the state using MAP, *s_MAP_*, can be represented as

(3) $$s_{MAP}=\underset{s_{i}\in State}{\arg\max}p(s_{i}|x_{SINR},x_{NI}),$$

where *s_i_* indicates the class within the possible states {*State*}, and *x_NI_* and *x_SINR_* represent the input values. By applying the Bayes’ theorem, we have

(4) $$s_{MAP}=\underset{s_{i}\in State}{\arg\max}\frac{p\left(x_{SINR},x_{NI}|s_{i}\right)}{p(x_{SINR},x_{NI})}\;=\underset{s_{i}\in State}{\arg\max}p(x_{SINR},x_{NI}|s_{i})p(s_{i}).$$

Assuming that the input values are conditionally independent, the outcome of the classifier, *s_MAP_*, can be calculated as

(5) $$s_{MAP}=\underset{s_{i}\in \{None,ShortIF,LongIF\}}{\arg\max}p(s_{i})p(x_{SINR}|s_{i})p(x_{NI}|s_{i}),$$

where *p*(*x_SINR_*| *s_i_*) and *p*(*x_NI_*| *s_i_*) can be obtained by observing the training data set.

**Figure 2:**
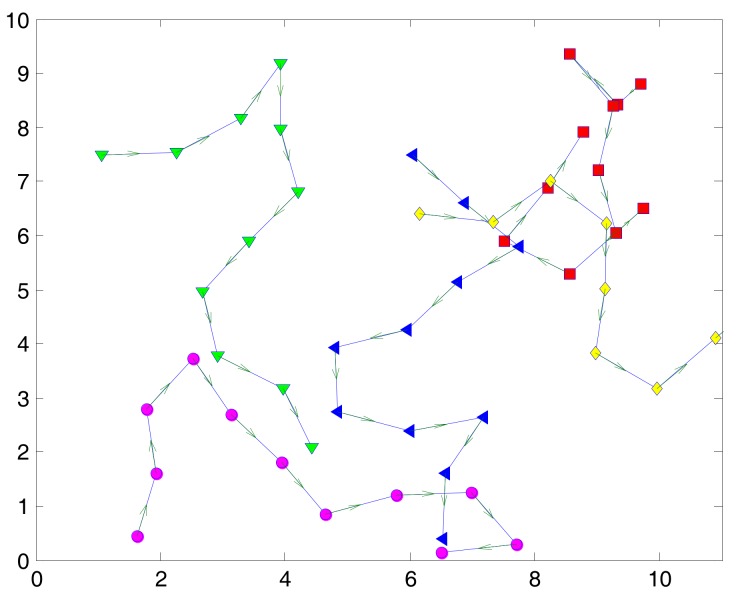
An example mobility scenario of five mobile WBANs.

**Figure 3:**
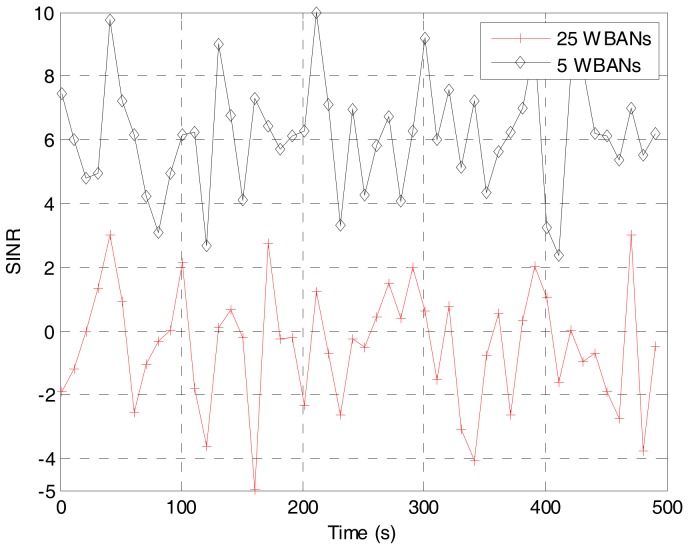
Average SINR in a WBAN for different numbers of WBANs in the deployment area.

**Figure 4:**
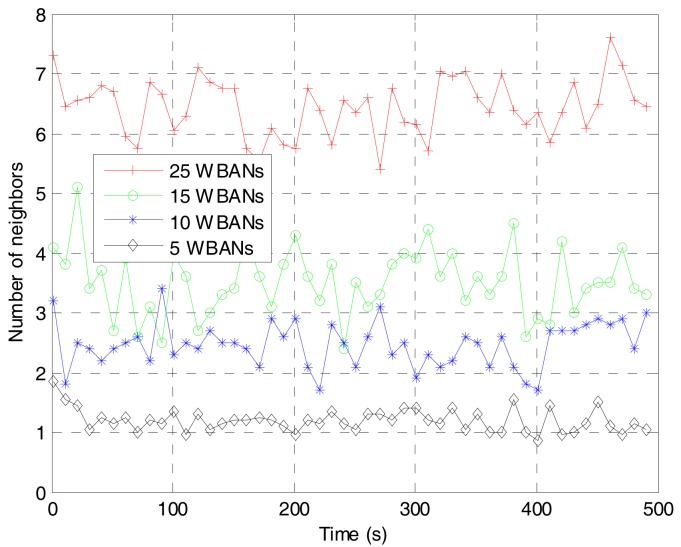
Average number of neighbors of a WBAN for different number of WBANs in the deployment area.

To verify the training value of the prediction algorithm, we deploy the network with multiple mobile WBANs for the duration of 100 s. The coordinator of each WBAN will follow the random waypoint model [32] to choose a destination at the next epoch time and move with a given speed while the sensor nodes follows the group velocity model. In our study, the deployment for multiple mobile WBANs is quite similar to the scenario for hospital applications in [33]. An example of the deployment area of five mobile WBANs is shown in Figure 2. A WBAN with an index number begins its movement at white signs which can be square, circle, or diamond marker, follows the dashed line as shown in the smaller marker, and ends at the black signs. Based on the deployment, we have extracted the average SINR and the average number of neighbors for a WBAN as shown in Figure 3 and Figure 4, respectively. From the extracted data, we have classified the inputs of the Bayesian inference classifier in Table 2. As a result, a training table is set up in Table 3 to predict the next state of the tagged WBAN. The threshold value (*T_thr_*) of interference duration is set as 10 s. 

In a real scenario for WBANs, a person can walk with low speed or high speed [8,33]. Therefore, we have evaluated the result of the interference prediction with the scenario of 25 WBANs moving with the speed of 0–2 m/s. For a single WBAN, the results of interference prediction are shown in Table 4.

## 4. Link Scheduling Algorithm Avoiding Interference in Multiple Mobile WBANs

At each epoch, the coordinator predicts the interference state for the next epoch based on the SINR, current status, and the number of neighbors. If the next status of a WBAN is *ShortIF*, the coordinator will broadcast a negotiation message to the network. Upon receipt of the messages from the neighbors, the coordinators exchange the TDMA schedule in the superframe for sharing the transmission channel in the interference avoidance process.

In each state of interference, the coordinator will perform the interference avoidance steps shown in Figure 1. First, the coordinator negotiates with its neighbors at the *ShortIF* state. Assume that the tagged WBAN *B_i_* is interfered with by a mobile WBAN *B_j_*. When *B_j_* moves into the interference range of *B_i_*, the coordinators of both WBANs require all the sensors to stop transmitting data. The coordinator of *B_i_* starts to negotiation for a shared superframe by broadcasting a HELLO signal. The coordinator of *B_j_* will send a REPLY signal because *B_j_* is in the transmission range of *B_i_*. The result of this step is to create a neighbor set *NB_i_*(*t*) for each WBAN. Then, the coordinators of *B_i_* and *B_j_* run the scheduling algorithm to allocate their data into fixed slots of the shared superframe.

### 4.1. MAC Superframe for Multiple WBANs

**Figure 5:**
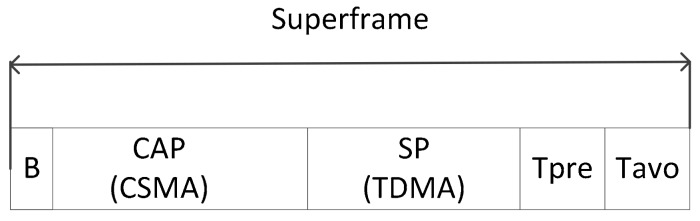
Superframe for multiple WBANs.

The scheduling algorithm is run on the WBAN *B_i_* subject to interference and its neighbors. The superframe structure for multiple WBANs is shown in Figure 5. A superframe consists of five parts: the beacon signal (B), the CAP, the SP, *T_pre_*, and *T_avo_*. In the CAP, the nodes in *NI_i_* perform CSMA/CA for intra-WBAN communication, which can be considered as the simple version of the exclusive access phase (EAP), random access phase (RAP), and CAP in an IEEE 802.15.6 MAC superframe. In the SP, the nodes in *I_i_* use TDMA for data delivery at the coordinator, which is similar to the managed access phase (MAP) in an IEEE 802.15.6 MAC superframe. The lengths of the CAP and the SP in our proposed superframe are adjusted to adapt to the number of interfered WBANs in *NB_i_*(*t*). *T_pre_* is the short duration for which *B_i_* predicts the next state based on the value of the SINR and the number of members in *NB_i_*(*t*). *T_avo_* is the short duration during which *B_i_* negotiates with every member in NB*_i_*(*t*) before starting the new superframe.

For intra-WBAN communication, the coordinators broadcast the result of scheduling to the sensor nodes via the beacon signal as shown in Figure 5. Therefore, the sensor node can transmit data to its coordinator via the fixed timeslot without interfering with the nearby WBANs.

**Figure 6:**
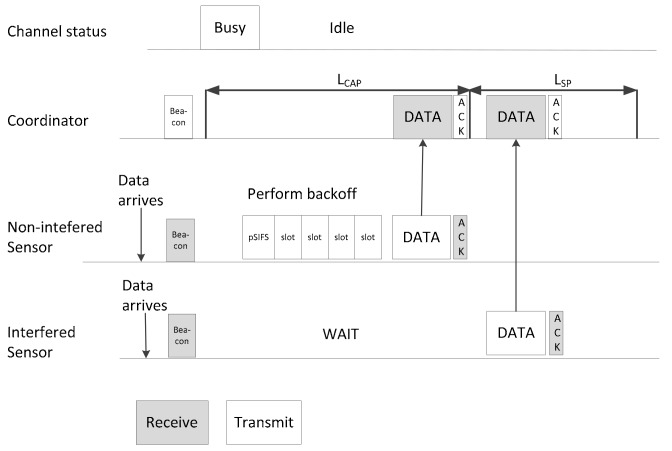
An example of intra-WBAN communication of interfered WBANs.

An example of transmission in the interfering WBANs is described in Figure 6. The non-interfering sensors will sense the channel, perform backoff, and start to transmit as if the channel is idle. The interfered sensor node only transmits at the assigned timeslot in the TDMA portion.

**Figure 7:**
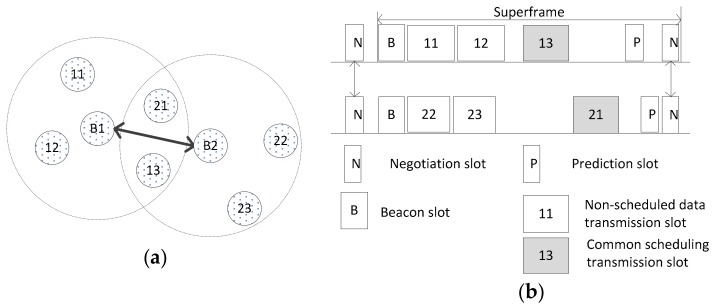
An example of inter-WBAN communication in two interfering WBANs: (**a**) negotiation between two WBANs; and (**b**) data transmission.

Figure 7 shows an example of inter-WBAN communication in two interfering WBANs. The transmission ranges of two WBANs *B*_1_ and *B*_2_ are overlapped, resulting in the interference of sensors 13 and 21 as shown in Figure 7a. The transmissions of sensors 13 and 21 are scheduled into the SP, which is called *common scheduling* among WBANs, as shown in Figure 7b. However, the signal transmission of the non-interfering sensors can occur in the CAP using CSMA/CA without a common scheduling.

### 4.2. Common Scheduling

At each WBAN, the lengths of the CAP and the SP are calculated for every epoch *T* because the number of members in *NB_i_*(*t*) changes. The length of the CAP is calculated by

(6) $$L_{CAP}=t_{s}\times \underset{i\in NB_{i}(t)}{\max}\left|NI_{i}\right|,NI_{i}=\{j|\gamma_{i}>\gamma_{th},d_{i,j}>R\}.$$

Likewise, the length of the SP is calculated by

(7) $$L_{SP}=t_{s}\times \sum_{i\in NB_{i}(t)}\left|I_{i}\right|,I_{i}=\{j|\gamma_{i}<\gamma_{th},d_{i,j}<R\}.$$

The required transmission time for a WBAN is calculated as

(8) $$T_{i}=\sum_{s=1}^{m}t_{s}=\sum_{s\in I_{i}}^{}t_{s}+\sum_{s\in NI_{i}}^{}t_{s},$$

where *t_s_* is the transmission time of a sensor node. Assuming that there are *k* neighbors of *B_i_*, so that |*NB_i_*| = *k*, the required transmission time, *T*(*k*), for *B_i_* and *NB_i_*(*t*) is calculated as follows:

(9) $$T(k)=\sum_{i=1}^{k}T_{i}=\sum_{i=1}^{k}\sum_{s\in I_{i}}^{}t_{s}+\underset{1\leq i\leq k}{\max}\sum_{s\in NI_{i}}t_{s}.$$

The length of a superframe for *k* interfering WBANs can be given as

(10) $$T_{ISF}=T(k).$$

Let *SF_m_* be the longest superframe that is acceptable for the latency of a vital signal. As in [27], if *T_ISF_* is longer than *SF_m_*, each coordinator will calculate the average number of slots for each WBAN.

The average number of time slots for each WBAN in the SP is calculated as

(11) $$T_{i}=\frac{T_{SPm}}{n},$$

where *T_SPm_* is the acceptable length of the SP that is calculated as

(12) $$T_{SPm}=SF_{m}-\sum_{i=1}^{n}\underset{s\in NB_{i}}{\max}\{t_{s}\}.$$

### 4.3. Negotiation and Scheduling Algorithm

**Figure 8:**
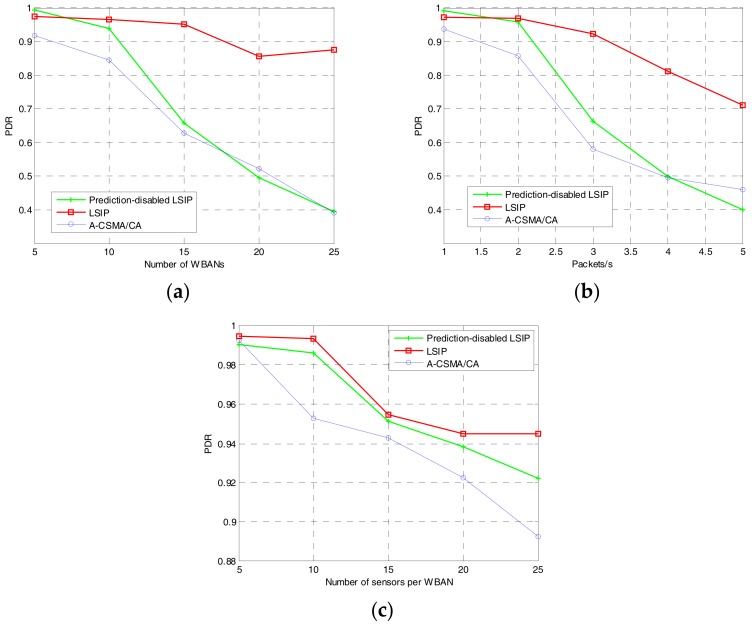
Packet delivery ratio: (**a**) varying the number of WBANs; (**b**) varying traffic load at each sensor node; and (**c**) varying the number of sensors per WBAN.

As per the flow chart in Figure 1, the coordinators will schedule the transmission after the prediction state. Figure 8 shows the link scheduling algorithm that is run at every coordinator of *V*(*t*) in multiple mobile WBANs. Inputs to the algorithm consist of the network topology, neighbor set, and resources of channels such as the maximum length of the superframe. The output of the algorithm is the schedule for each WBAN into a hybrid MAC superframe. For the CAP part, the non-interfering sensors will sense the channel and perform backoff before transmitting data to the coordinator. The interfering sensors must wait until their assigned timeslot in the TDMA portion to transmit data.

The algorithm proceeds in two phases. First, calculate the length of the CAP and the SP. Next, assign the timeslot to every node transmission according to the traffic at the sensor node. Each WBAN calculates a contention value *s_i_*(*t*) for scheduling by using the total number of interfering sensors and the number of neighbors as follows:

(13) $$s_{i}(t)=\frac{\sum I_{i}}{M}\times \frac{\sum NB_{i}(t)}{\sum NB_{i}(t-1)}$$

In the link scheduling algorithm shown in Algorithm 1, at the first phase, each coordinator will broadcast the list of interfering sensor nodes to its neighbors (line 1). After sending and receiving this list, the coordinators create two common lists of sensor nodes which are the interfering and non-interfering sensor nodes at the current time *t*, denoted as *CI*(*t*) and *CNI*(*t)*, respectively (lines 2 to 5). Each coordinator will calculate the length of *L_CAP_*, *L_SP_*, and *T_ISF_* (line 6). The total length of the superframe is calculated (line 7). In the case the length of the superframe exceeds the acceptable length *SF_m_*, the lengths of SP and CAP are calculated as in lines 8–12. At the second phase, the first available time slot is assigned to the sensor node with the highest priority in *CI*(*t)* (line 15). Therefore, all the sensors in *CI*(*t)* will be assigned to a time slot in the superframe.

**Algorithm 1:** LSIP algorithmInput: *NB_i_*(*t*), *SF_m_*, *I_i_*, *NI_i_*, *t_s_*Output: scheduled superframeInitialize: *t* = 0// **Phase 1: Calculate length of superframe**1.  *B_i_* broadcasts {*I_i_*, *NI_i_*} to all members in *NB_i_*(*t*)2.  **For** each *C_j_* ∈ *NB_i_*(*t*)3.    Receive {*I_j_*, *NI_j_*}4.    Create a common list of neighbors: *CI*(*t*) = {*s_j_*(*t*) ∪ *s_i_*(*t*), *I_i_* ∪ *I_j_* | j ∈ *NB_i_*(*t*), *i* ∈ *NB_j_*(*t*)}, *CNI*(*t*) = {*NI_i_* ∪*NI_j_* | *j* ∈ *NB_i_*(*t*), *i* ∈ *NB_j_*(*t*) }**5**.  **End For**6.  Calculate *L_CAP_* and *L_SP_* as in (6) and (7), respectively7.  *T_ISF_* = *L_SP_* + *L_CAP_*8.  **If**
*T_ISF_* > *SF_m_*9.      Calculate *TSP_m_* as in (12)10.    Calculate *T_i_* as in (11)11.    Update *L_SP_* = *T_SPm_***12**.  **End If**// **Phase 2: TDMA scheduling by using greedy algorithm**13.  **For** each sensor *s* ∈ *CI*(*t*)14.    Return the sensor *s_x_* with the highest contention value in *CI*(*t*)15.    Assign time slot to *s_x_* with length *t_s_* (*t_s_* is transmission time of *s_x_*)16.    Update *t* = *t* + *t_s_*17.    Remove *s* of *CI*(*t*)18.    **If**
*t* > *L_SP_*19.     break **20**.    **End If****21.** **End For**

## 5. Performance Evaluation

In this section, we conduct a performance evaluation via Matlab simulations for our proposed LSIP. We also compare this performance with the A-CSMA/CA system in [28]. The interference mitigation algorithm in [28] reduces inter-WBAN interference by using CSMA at the coordinator and a hybrid superframe for intra-WBAN transmission.

### 5.1. Simulation Environment

In our simulation, the scenario of WBANs for health monitoring is assumed, where many people are wearing WBANs at the same time, as in [33]. The mobility scenario for simulation is similar to the mobility scenario in Figure 2 in Section 2. The velocity of WBANs is set as 0–1.5 m/s, according to [8,33]. We executed the simulations for 500 s with different sets of parameters to evaluate the density of the network and the traffic load in each WBAN. To evaluate the dense deployment of the network, we vary the number of WBANs and the number of sensors per WBAN. Each WBAN consists of one coordinator and several sensors nodes with the transmission range of 2 m [2]. The traffic load for WBANs is assumed to the number of packets generated by sensor nodes. Varying the number of generated packets per second at each sensor node simulates the traffic load for each WBAN. The power consumption at the coordinator is set as 31.2 mW for transmission and 27.3 mW for reception as in [27]. Simulation parameters and their values used in the simulation are shown in Table 5. We only consider the flexibility and reliability of our scheduling algorithm in the mobile scenario so that we do not consider the priority of sensor nodes. However, we evaluate the scheduling algorithm in the worst case where all the sensor nodes are in the overlapped area with neighboring WBANs. For that matter, the CAP in a superframe is not implemented in the simulation; the transmissions from sensor nodes to the coordinator are allocated in the SP by the proposed scheduling algorithm.

We also simulate different scenarios with and without interference prediction. With interference prediction, each WBAN will transmit and schedule according to the proposed algorithm in Figure 1. Without interference prediction, the coordinator broadcasts the negotiation message at the end of each epoch time. We measure the four performance metrics: packet delivery ratio, end-to-end delay, network throughput, and energy consumption per bit by varying three different parameters. First, we vary the number of WBANs in the network, in which each WBAN consists of 10 sensor nodes and each sensor node generates two packets per second. Next, we vary the number of generated packets per sensor node, in which there are 10 WBANs in the network area and each WBAN consists of 10 sensor nodes. Lastly, we vary the number of sensor nodes per WBAN, in which the number of WBANs is 10 and each sensor node generates two packets per second.

### 5.2. Simulation Results and Discussion

#### 5.2.1. Packet Delivery Ratio

Packet delivery ratio (PDR) is calculated as the number of data packets received at the coordinator over the number of actual generated packets at the sensor nodes. It is shown in Figure 8 that the proposed algorithm with interference prediction achieves better performance than the other methods. In dense networks and/or with high traffic load, PDR decreases for all three methods. However, our proposed algorithm outperforms the others for all cases. Due to the common scheduling and interference prediction steps, the sensor nodes transmit data during the allocated time slot that does not interfere with any transmission from neighbors. In the LSIP, therefore, the number of the data packets received at the coordinator is increased. In the prediction-disabled LSIP, however, the common scheduling is not updated when a new neighboring WBAN moves closer. It results in interference with the new neighboring WBAN.

#### 5.2.2. End-to-End Delay

**Figure 9:**
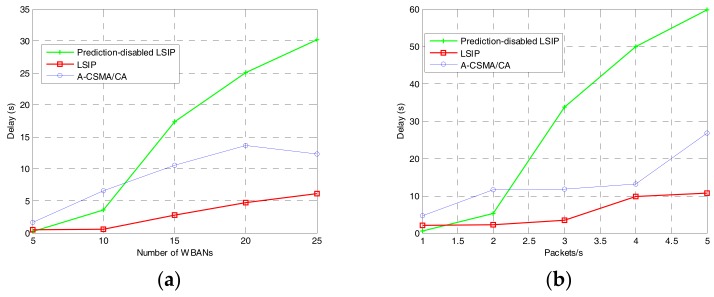

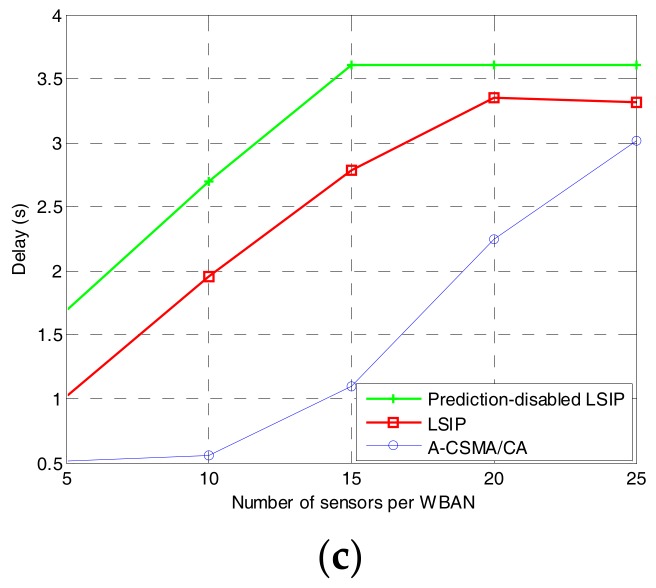
End-to-end delay: (**a**) varying the number of WBANs; (**b**) varying traffic load at each sensor node; and (**c**) varying the number of sensors per WBAN.

End-to-end delay is calculated as the average duration from the time when a packet is generated at a sender node to the time when the packet is received at the coordinator. The simulation results of the end-to-end delay are shown in Figure 9. In three scenarios, when the traffic load or network density increases, the end-to-end delay also increases. More specifically, due to the prediction and negotiation steps, the end-to-end delay of our proposed algorithm is higher than A-CSMA/CA when we vary the number of sensors per WBAN. However, in the worst case for high density and high traffic load, our proposed algorithm achieves lower end-to-end delay. In the case without interference prediction, a WBAN takes more time for negotiation because the neighbor set of WBANs changes over time. In the case with interference prediction, however, a WBAN only negotiates with the specific neighbors, which results in lower delay.

#### 5.2.3. Network Throughput

**Figure 10:**
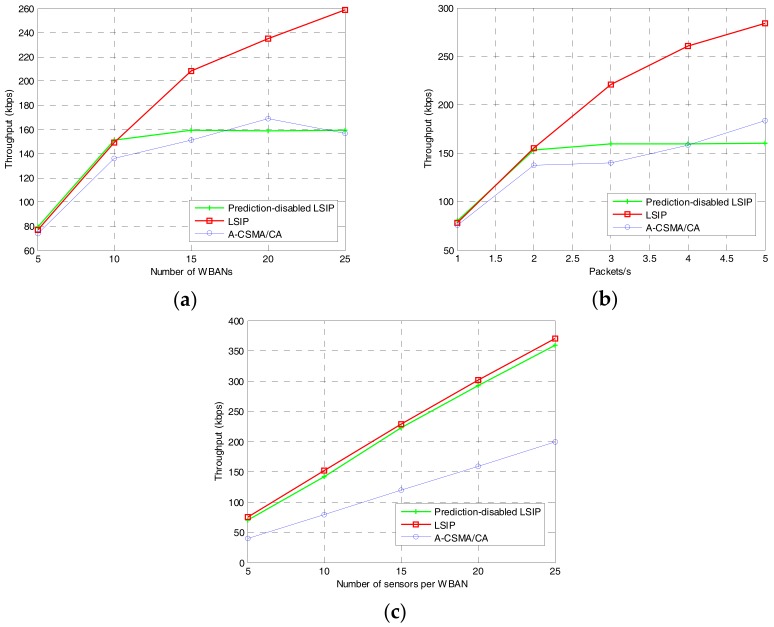
Network throughput: (**a**) varying the number of WBANs; (**b**) varying traffic load at each sensor node; and (**c**) varying the number of sensors per WBAN.

Network throughput is the average rate of successful packet deliveries at the receiver, which is usually measured in bits per second or packets per second. In our evaluation, we calculate the network throughput in kilobits per second (kbps) for the data packets received at the coordinator. The results are shown in Figure 10 in three scenarios. Our proposed algorithm outperforms the other methods. The network throughput depends on the density of the network or the traffic load of the sensor node. As in Figure 10a,b, without interference prediction, the network throughput of our proposed algorithm is similar to that of A-CSMA/CA. Even though the proposed algorithm allows multiple WBANs to transmit at the same time by using the shared superframe, due to lack of interference prediction, the coordinator takes more time slots for finding the neighbors for negotiation. With interference prediction, the superframe can be kept a long time because the neighbor set of a WBAN may not change, resulting in higher throughput for the proposed algorithm.

#### 5.2.4. Energy Consumption

**Figure 11:**
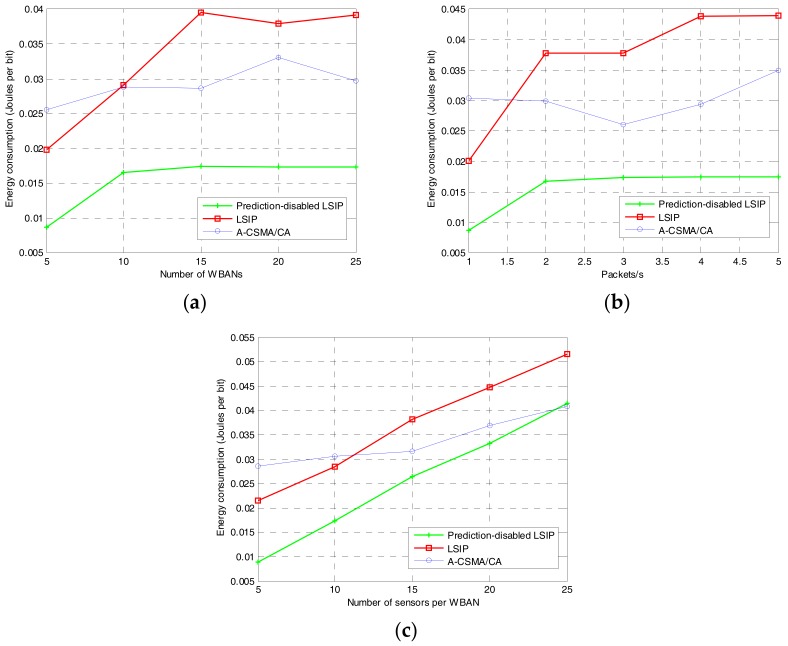
Energy consumption at the coordinator: (**a**) varying the number of WBANs; (**b**) varying traffic load at each sensor node; and (**c**) varying the number of sensors per WBAN.

Energy consumption of a coordinator is calculated in Joules per bit. At the coordinator, power consumption is the amount of power consumed at different states, which are: (a) the transmitting state, where the coordinator transmits the beacon and negotiation messages; (b) the receiving state, where the coordinator receives data packets from sensor nodes and negotiation messages; and (c) the interference prediction states. It should be noted that, after receiving the beacon from the coordinator, the sensor nodes transmit the sensed data according to the information included in the beacon and then enter the sleep mode. As shown in Figure 11, the power consumption of the proposed algorithm depends on the traffic load and the network density whereas that of A-CSMA/CA varies in a range, but does not depend on the traffic load or the network density. In the proposed algorithm, however, the power consumption also depends on the duration and negotiation messages where the coordinator negotiates the schedule with its neighbors. Therefore, the coordinator consumes more energy when the network density or the traffic load increases. It should be noticed that the energy consumption in our simulation study contains the energy consumed at the coordinators of multiple WBANs and, in a WBAN, the coordinator consumes much more energy than sensor nodes. Typically, the coordinator is equipped with high-capacity battery in comparison to the sensor nodes. Therefore, even though the proposed algorithm consumes more energy, the energy consumption is not a critical issue in the most applications of multiple mobile WBANs. Instead, both the reliability and the throughput, which are very important and sometimes critical in the target applications of health monitoring, are significantly improved in comparison to the other algorithms as shown earlier. On the other hand, the proposed algorithm without interference prediction consumes less energy than A-CSMA/CA because the coordinator does not perform sensing channel and back off for channel contention.

## 6. Conclusions

In this paper, we have proposed a link scheduling algorithm with interference prediction for multiple mobile WBANs. We have shown that the Bayesian inference classifier, which is simple and has a low computational complexity, can be easily deployed to predict the interference state of WBANs. With interference prediction, a WBAN can obtain knowledge about its current state and its neighbor set, which results in a short link schedule amongst nearby WBANs. In addition, common scheduling is also proposed, which allows for multiple concurrent intra-WBAN transmissions without interference. We have also proposed a method to calculate the contention value for each WBAN in the common scheduling considering the interference level of WBANs, enabling the signal transmission of a WBAN even with interference. The proposed LSIP improves the packet delivery ratio and network throughput remarkably with acceptable delay by overcoming the inter-WBAN interference in comparison to the conventional scheme. Nonetheless, the negotiation between the WBAN coordinators requires additional energy consumption. As a future work, we will investigate the development of more energy-efficient negotiation mechanisms. As another possible future work, we are going to consider coexistence among WBANs including the mmWave technology.

## Figures and Tables

**Table 1:** Defined coexistence environment.

Environment	Description
Static (S)	A single WBAN in a residential environment or a hospital with a single patient node and a fixed bedside hub.
Semi-dynamic (SD)	Slowly moving ambulatory patients in an elder care facility requiring infrequent and/or event-based low-rate data transfers.
Dynamic (D)	Fast moving ambulatory patients in a hospital with several WBANs collecting continuous data traffic from many sensor nodes.

**Table 2:** Classification table.

Category	No Interference	Short Time	Long Time
Duration of interference	0	<10 s	>10 s
Number of neighbors	<2 neighbors	2–6 neighbors	>6 neighbors
SINR	>6 dB	1–6 dB	<1 dB

**Table 3:** Training table.

SINR	Number of Neighbors	Previous State	Current State
1–6 dB	<2 neighbors	Short IF	No IF
1–6 dB	<2 neighbors	Long IF	Short IF
1–6 dB	2–6 neighbors	Short IF	Short IF
1–6 dB	2–6 neighbors	Long IF	Long IF
1–6 dB	>6 neighbors	Short IF	Long IF
1–6 dB	>6 neighbors	Long IF	Long IF
>6 dB	2–6 neighbors	No IF	No IF
>6 dB	2–6 neighbors	Short IF	No IF
>6 dB	2–6 neighbors	Long IF	Short IF
>6 dB	>6 neighbors	No IF	Short IF
>6 dB	>6 neighbors	Shor IF	Long IF
>6 dB	>6 neighbors	Long IF	Long IF

**Table 4:** Results of interference prediction.

Scenario	Current State	Next State
SNIR = 2.0735; Deg = 5; Previous_state = noIF	Short IF	Short IF
SNIR = 0.65; Deg = 6; Previous_state = ShortIF	Long IF	Long IF
SNIR = −2.6; Deg = 5; Previous_state = LongIF	Long IF	Long IF
SNIR = −4; Deg = 9; Previous_state = ShortIF	Long IF	Long IF
SNIR = 11; Deg = 7; Previous_state = LongIF	Short IF	Short IF

**Table 5:** Simulation parameters.

Parameters	Value
Number of WBANs	5–25 (default: 10)
Number of sensors per WBAN	5–25 (default: 10)
Simulation area	10 m × 10 m
Transmission range	2 m
Velocity of each WBAN	0–1.5 m/s
Direction of motion vector	Random
Simulation time	500 s
Slot allocation length	10 s
Negotiation time	10 ms to *N* × 10 ms
Packet size	100 bytes
Packet transmission rate	1–5 packets/s (default: 2)
Tx power consumption	31.2 mW
Rx power consumption	27.3 mW
Data rate	250 kbps
